# Patient-Reported Outcomes with Peripheral Nerve Stimulation for Low Back Pain from Vertebral Plana Deformities: A Case Series

**DOI:** 10.3390/jcm14113964

**Published:** 2025-06-04

**Authors:** Saba Javed, Loc Lam, Angela Nwankwo, Zaur Komachkov

**Affiliations:** 1 Department of Pain Management, University of Texas MD Anderson Cancer Center, Houston, TX 77030, USA; sjaved@mdanderson.org; 2 H. Ben Taub Department of Physical Medicine and Rehabilitation, Baylor College of Medicine, Houston, TX 77030, USA; angela.nwankwo@bcm.edu (A.N.); zaur.komachkov@bcm.edu (Z.K.)

**Keywords:** peripheral nerve stimulation, cancer pain, plana fracture, temporary PNS, neuromodulation, acute pain, chronic pain

## Abstract

**Objectives:** This study evaluated peripheral nerve stimulation (PNS) as a treatment for vertebra plana fractures, focusing on its impact on pain intensity, physical function, anxiety, depression, fatigue, social role participation, and pain interference. The goal was to assess whether PNS could serve as a minimally invasive alternative for managing pain in patients with severe vertebral fractures. **Methods:** Four patients with lumbar vertebra plana fractures received PNS implants for 60 days. Pain scores and PROMIS-29 domains (physical function, anxiety, depression, fatigue, social participation, and pain interference) were assessed at 30, 60, 90, 180, and 365 days post-implantation. Data analysis included mean and standard deviation calculations. **Results:** PNS led to marked improvements in pain-related outcomes. The average pain intensity scores dropped from 8.5 at baseline to 4.25 at one year, and pain interference scores declined from 61.75 to 54.75. Physical function initially decreased but improved from 38.5 at three months to 46.75 at one year. Changes in depression, anxiety, fatigue, and social participation were minimal, reflecting their multifactorial nature and limited response to pain relief alone. **Conclusions:** This case series suggests that PNS may significantly reduce pain and pain interference while enhancing physical function in patients with vertebra plana fractures. Its sustained benefits highlight PNS as a promising minimally invasive treatment, especially for those ineligible for traditional procedures. However, the limited improvement in psychological and social domains underscores the need for comprehensive care strategies. Further research is warranted to explore the broader role of PNS in managing vertebral fracture pain.

## 1. Introduction

Vertebral compression fractures (VCFs) are the most common fragility fracture afflicting the elderly. In 2001, the annual cost of VCF management was an estimated USD 746 million [1]. With an aging population, the number of VCFs is expected to increase. In addition to trauma and malignancy, osteoporosis is the most frequent cause, with over 10 million elderly patients diagnosed each year [1]. Although the fractures typically heal on their own after a few months, they are associated with increased morbidity and mortality [2].

Pain from VCFs is multifactorial in origin, and understanding its mechanisms is essential for selecting appropriate interventions. Specifically, nociceptive pain arises from mechanical instability and inflammation resulting from vertebral collapse and altered spinal biomechanics [3]. Neuropathic pain may result from the direct or indirect irritation of neural structures, including the medial branch nerves and basivertebral nerves, due to vertebral deformation or inflammation [3]. In more chronic presentations, patients may also experience nociplastic pain, characterized by central sensitization and amplified pain perception despite minimal ongoing tissue damage [4]. These overlapping pain pathways contribute to persistent disability and make treatment complex.

The pain from VCFs can hinder mobility, which leads to physical deconditioning and increased mortality even after the fractures have healed. Therefore, the goal of treatment should be to control pain to enable the retention of functional capacity [5,6]. Current treatment options for VCF pain include medications, physical therapy, and bed rest. Minimally invasive approaches may involve radiofrequency ablations. Advanced procedures, such as vertebroplasty and kyphoplasty, are also available. Spine surgery is typically reserved for cases with neurologic compromise [7,8,9,10].

While the percutaneous augmentation of the vertebrae is relatively safe in a typical compression injury, the procedures become dangerous if attempted in vertebral plana fractures due to difficulty entering the pedicle and the high risk of cement extravasation. PNS is a promising treatment for acute lower back pain due to several etiologies, including VCFs [11]. PNS provides the targeted stimulation of the peripheral nerves and can be temporary or permanent. It has also been used in the management of refractory cancer pain, postoperative pain in total knee arthroplasty [12,13], headaches, complex regional pain syndrome, and low back pain [14,15]. In a recent study, a 60-day percutaneous PNS placement provided lasting relief for lower back pain, demonstrating how PNS helps reduce “arthrogenic inhibition” and “post-traumatic hypersensitivity”, which are common causes of chronic pain [16]. Gilligan et al.’s research on restorative neurostimulation in multifidus dysfunction highlights the relevance of PNS for lower back pain associated with muscle instability, reinforcing its application in conditions like VCFs [17]. Together, these findings underline PNS as a versatile and effective modality in managing acute and chronic pain.

PNS may be particularly useful for posterior VCF pain, which results from the vertebral body collapse, causing a shift in the posterior elements, such as facet joints, due to altered biomechanics and compensatory stress [18]. Facet-mediated pain is driven by the irritation of the medial branch nerves and contributes to persistent posterior VCF discomfort. Radiofrequency ablation (RFA) provides alleviation by denervating medial branch nerves but also results in the possible atrophy of multifidus [16]. PNS offers a minimally invasive alternative that targets medial branch nerves and relieves facet-mediated pain while preserving medial nerve function, unlike RFA. PNS provides an approach to managing posterior VCF pain and enhancing functional recovery [16].

Herein, we present a unique series of four patients with severe pain due to vertebral plana fractures who underwent temporary PNS and were followed for 12 months with patient-reported outcomes (PROs). To our knowledge, this is the first case series to longitudinally evaluate PROs via ROMIS-29 following temporary medial branch-targeted PNS in patients with vertebra plana fractures. This population presents a therapeutic challenge due to the high procedural risk of vertebral augmentation and the multifactorial nature of pain, which includes nociceptive, neuropathic, and nociplastic components. Unlike radiofrequency ablation, which may result in paraspinal muscle atrophy, PNS preserves medial branch function and supports muscular integrity. Furthermore, PNS offers a non-opioid, minimally invasive, anesthesia-sparing treatment path for patients who are often elderly or medically complex. By characterizing functional, emotional, and social domain outcomes over 12 months, this study provides new insights into the role of neuromodulation in managing complex vertebrogenic pain where traditional interventions are unsuitable.

## 2. Materials and Methods

Patient characteristics are summarized in Table 1 below.

This case series included four patients with lumbar vertebra plana fractures observed between 2020 and 2023. The diagnosis was based on radiographic criteria, defined as a greater than 70% reduction in anterior vertebral height compared to the posterior column.

Inclusion criteria were as follows:

- Age over 18 years.
- Evaluation at a pain management clinic.
- Radiographically confirmed vertebra plana fracture with >70% vertebral height loss.
- Persistent pain despite standard conservative management.

Exclusion criteria included the following:

- Patient’s refusal to participate or to complete a 90-day follow-up.
- Inability to undergo percutaneous PNS due to active infection, severe coagulopathy, or anatomical contraindications.

Prior to considering PNS, patients underwent standard clinical treatments, including management with opioids, nonsteroidal anti-inflammatory drugs, physical therapy, and bracing. All treatments failed to provide adequate relief, leading to the implementation of PNS.

Patients were implanted with temporary PNS systems for 60 days. Stimulation was delivered with initial standard settings of 12 Hz frequency and six-hour timed sessions. Therapy was applied for up to 12 h daily, depending on patient tolerance and symptom response. Parameters were adjusted within manufacturer-recommended ranges to optimize clinical outcomes: pulse width 10–200 microseconds (μs), amplitude 0.2–30 milliamperes (mA), and frequency 5–150 Hz, using the Hand-Held Remote or Clinical Programmer interface as needed. Stimulation intensity was titrated in increments of 2 units, and amplitude was increased gradually to evoke either a comfortable paresthesia or visible multifidus contraction during initial calibration. Adjustments over the treatment course were individualized, based on patient-reported outcomes and tolerability.

Pain intensity and functional outcomes were evaluated using the PROMIS-29 domains. Assessments were conducted at baseline and at 30, 60, 90, 180, and 365 days post-treatment.

The NIH Patient-Reported Outcomes Measurement Information System (PROMIS) 29 is a standardized tool developed by the National Institutes of Health to measure patient-reported outcomes across a wide variety of chronic conditions, including chronic pain [19]. The PROMIS-29 includes 29 items across seven domains: Physical Function, Anxiety, Depression, Fatigue, Sleep Disturbance, Ability to Participate in Social Roles and Activities, and Pain Interference. Additionally, it includes a single item on pain intensity. Its utility lies in capturing the multifaceted nature of chronic pain and its impact on various aspects of daily life. Validation studies have shown that PROMIS-29 is both reliable and sensitive in detecting changes in pain levels, physical function, and emotional distress among patients with chronic pain [20]. The tool has been widely adopted in clinical and research settings due to its ability to provide a comprehensive assessment of patient well-being and track interventions’ outcomes over time.

This study was conducted with Institutional Review Board approval, ensuring ethical adherence and patient consent. The radiographic diagnosis of vertebral plana deformities was confirmed using plain radiographs and magnetic resonance imaging (MRI). These modalities were used to assess vertebral height loss and the structural integrity of the anterior and posterior elements.

Temporary PNS systems were utilized, with stimulation provided continuously or intermittently based on patient needs. Electrodes were placed percutaneously, and stimulation settings were optimized to provide maximum pain relief. Treatment lasted for 60 days, with devices removed after this period to assess the durability of pain relief and functional improvement.

## 3. Case Presentations

No complications occurred during the placement of the PNS devices. All systems were placed percutaneously under fluoroscopic guidance using local anesthesia, minimizing patient discomfort and reducing the risks associated with more invasive procedures. Mild discomfort at the electrode site was reported in two patients, which resolved within a few days without requiring additional intervention. There were no instances of infection, device migration, or hardware failure observed throughout the study period or upon the removal of the devices after the 60-day treatment period. The specific target of the leads was the medial branches of the nerve roots at the level of the injury to stimulate the multifidus muscles. In our first patient, we were unable to thread the leads to the level of the injury due to anatomic difficulties, so leads were placed at the level below. In the other three patients, leads were placed at the initial target.

### 3.1. Case 1

**Figure 1:**
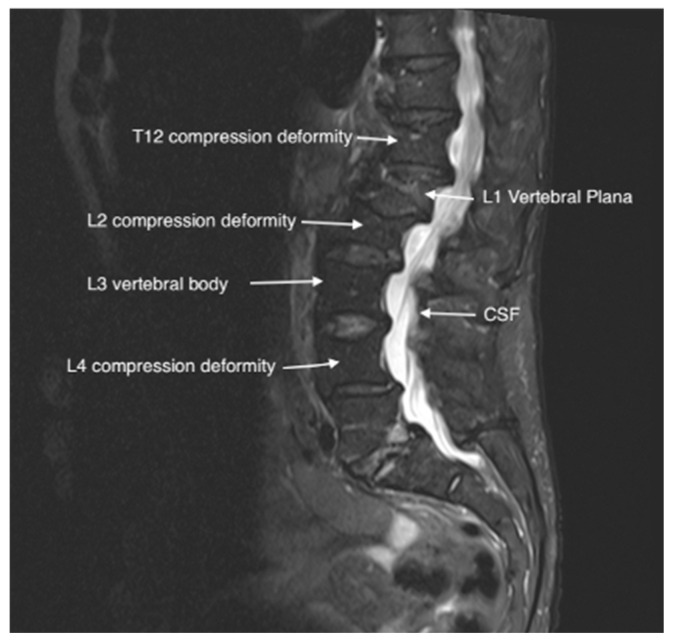
T2 imaging of L4 vertebral plana fracture in Case 1.

**Figure 2:**
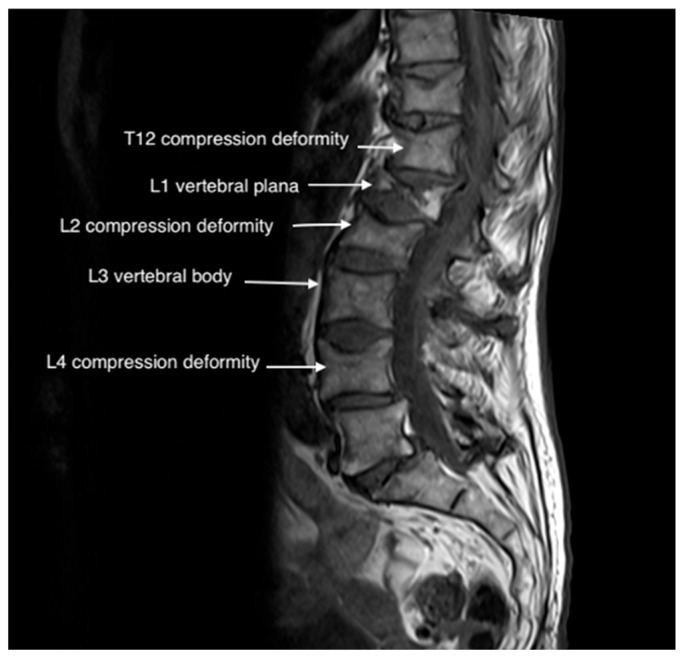
T1 imaging of L4 plana fracture in Case 1.

An 80-year-old female with a history of coronary artery disease (CAD), cardiac arrest, and squamous cell carcinoma of the tongue presented to the pain clinic with worsening low back pain after a fall. Her pain was rated 9/10, dull, and persistent, with no radiation to the lower extremities. She was mostly sedentary due to pain, and acetaminophen 650 mg three times daily was ineffective. A physical exam revealed tenderness around T12-L2 and positive facet loading. MRI confirmed an L1 vertebral plana fracture, T12-L2 fractures, and metastatic lesions. Neurosurgery was consulted but did not recommend intervention. Due to concerns about steroids and anesthesia, the patient declined facet injections and vertebral augmentation but agreed to PNS under local anesthesia (Figure 1 and Figure 2).

**Figure 3:**
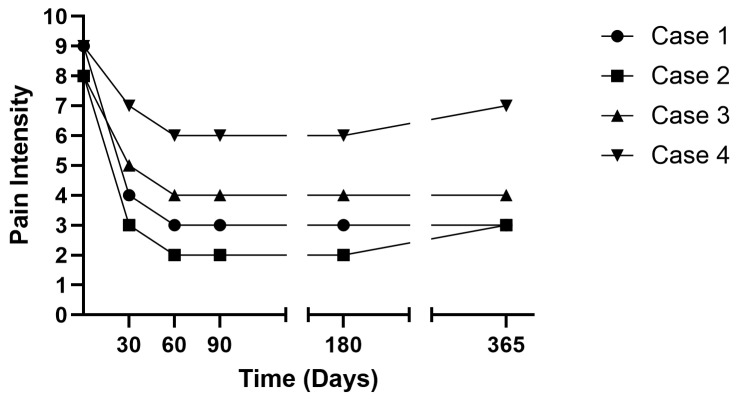
Changes in pain score over time.

**Figure 4:**
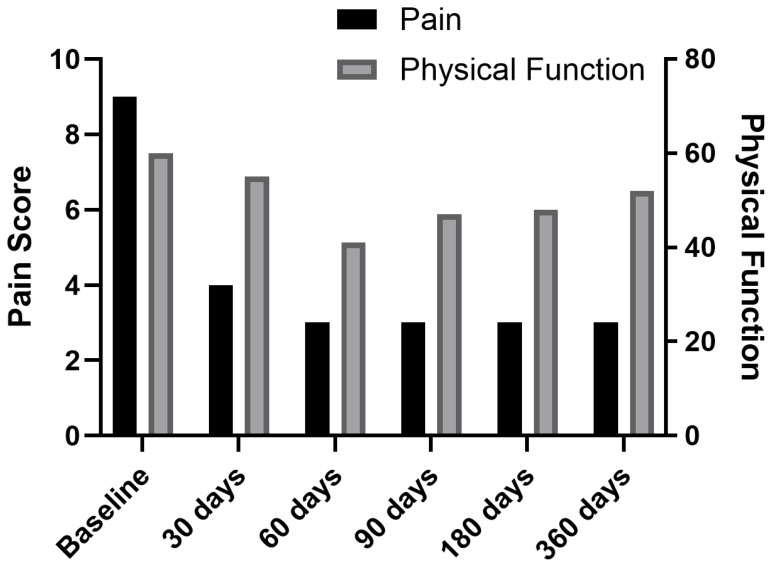
Change in pain scores and physical function scores over time for Case 1.

Under fluoroscopic guidance, electrodes were placed bilaterally at T12 medial branch (target 1 cm medial and inferior to the facet joint). We induced a stimulus of 12 Hz frequency and amplitude of 0.1–30 mA with a ramped waveform to elicit the contraction of the muscles, confirming electrode placement. The duration of the charge was 200 μs using a fast cycle on/off ratio of 1 s each. After the initial charge, we adjusted the level of stimulation to adhere to patient relief. Immediately post-procedure, the patient’s pain decreased to 7/10. At 1-week follow-up, pain further reduced to 5/10, with decreased opioid use. By 1 month, she discontinued opioids entirely and reported 4/10 pain. At her 30, 60, 180, and 365-day follow-ups, pain remained at 3/10 without opioid use (Figure 3 and Figure 4).

Over the 12-month follow-up, this patient exhibited sustained pain relief and complete opioid cessation by day 30. PROMIS-29 physical function improved moderately, while psychological and social scores remained essentially unchanged, reflecting persistent limitations in daily engagement. No therapy modifications were needed post-PNS.

### 3.2. Case 2

**Figure 5:**
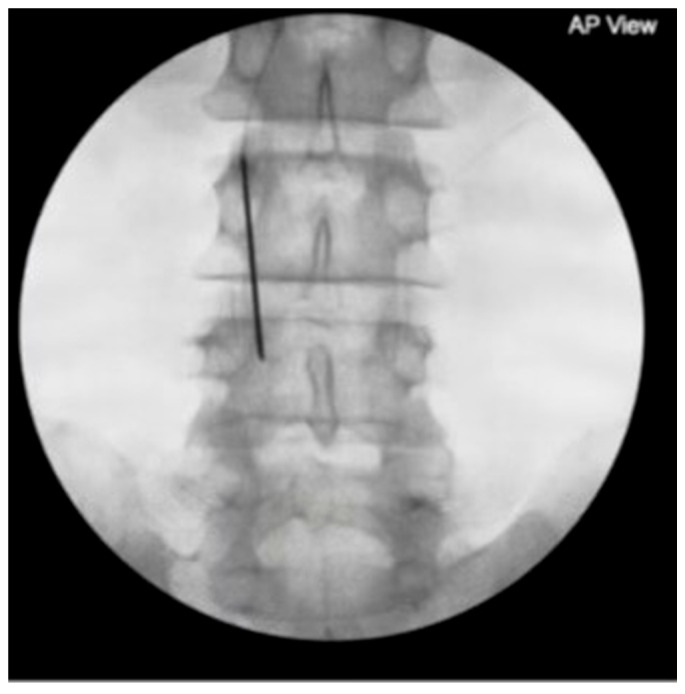
Fluoroscopic image of PNS needle tip at L4 for L4 placement targeting bilateral L3–L4 and L4–L5 medial nerve branches in Case 2.

A 71-year-old male with a history of diabetes mellitus (DM), rheumatoid arthritis, and degenerative disk disease presented with newly diagnosed multiple myeloma (MM) and an L4 vertebral plana fracture secondary to metastatic lesions. He had persistent, dull, achy pain for one month, aggravated by standing. A physical exam showed lumbar tenderness and positive facet loading. MRI revealed an L4 compression deformity and interventional radiology planned radiation therapy. Initial pain management included gabapentin and oxycodone 5 mg, which he took 4–5 times daily. PNS and lumbar facet injection were discussed, and he opted for fluoroscopy-guided bilateral PNS at the L3 medial branch. Figure 5 exhibits a needle at the L4 vertebral body. The same stimulation parameters were used as in the previous case.

**Figure 6:**
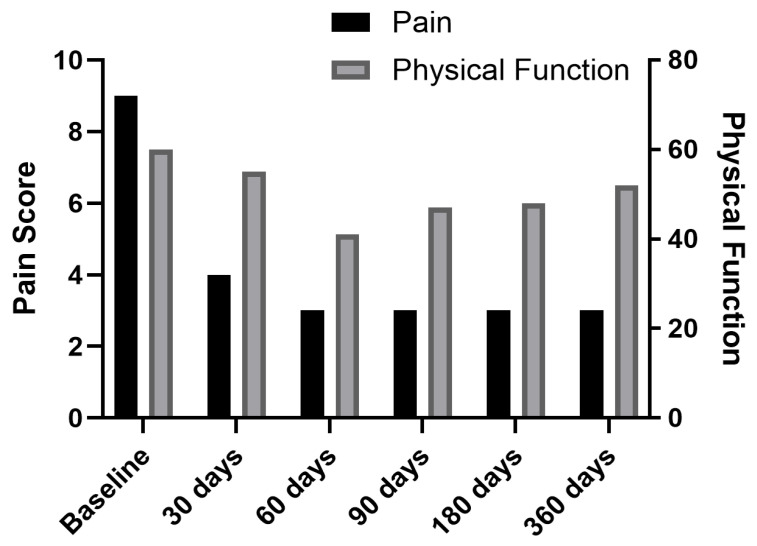
Change in pain scores and physical function scores over time for Case 2.

Postoperatively, his pain improved from 8/10 to 4/10 by the next day. At the 30-day follow-up, pain was 3/10, and by 60 days, it was 2/10. Leads were removed at 60 days, which is at the same time when he completed radiation therapy, and oxycodone use decreased to twice daily. Pain remained 2/10 at 90-day and 6-month follow-ups. At 360 days, pain was reported as 3/10 (Figure 3 and Figure 6).

This patient showed rapid pain reduction post-PNS with durable relief through 1 year, even after radiation therapy. Functional gains were stable, though psychological domains (anxiety/depression) showed minimal change. Opioid use was significantly reduced but not fully discontinued. Social activity remained limited, likely due to disease burden.

### 3.3. Case 3

An 85-year-old female with a history of carotid artery stenosis, CAD, hypertension, DM, osteoarthritis (OA), osteoporosis, and subacute VCF at multiple levels presented with worsening low back pain. Her acute-on-chronic pain was attributed to osteoporotic fractures at T10, T12, L2, and L3, with worsening mid-lower back pain. A physical exam showed lumbar tenderness and positive facet loading. Initial pain management included acetaminophen and hydrocodone/acetaminophen, but facet injections at L2/3 and L3/4 provided minimal relief.

**Figure 7:**
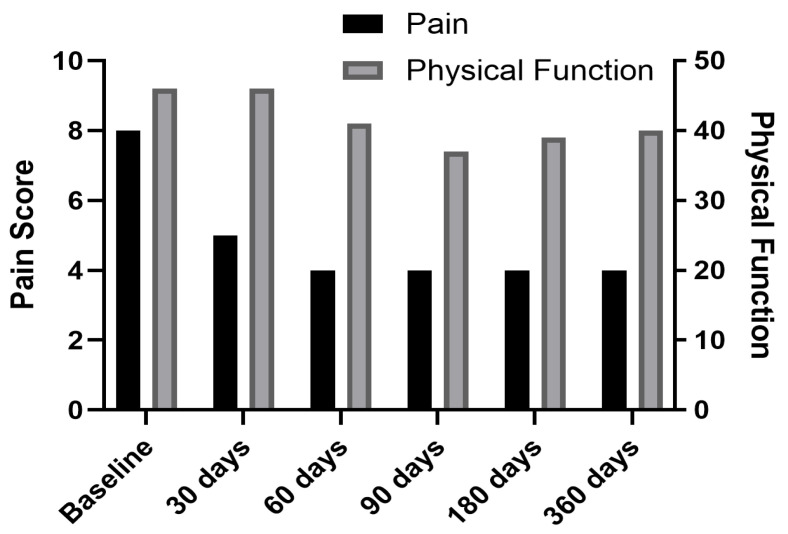
Change in pain scores and physical function scores over time for Case 3.

Two weeks later, fluoroscopy-guided PNS was performed at L3 and L2 medial branch bilaterally, utilizing the same stimulation parameters as those in the previous cases. Postoperatively, pain decreased from 8/10 to 7/10 on day one. At 30 days, pain was 5/10, and by 60 days, it further decreased to 4/10. Leads were removed at 60 days, and hydrocodone/acetaminophen use was reduced. Pain relief remained stable at 4/10 at 90, 180, and 360-day follow-ups (Figure 3 and Figure 7).

Despite multiple comorbidities and widespread vertebral disease, this patient experienced a gradual but steady decline in pain scores with stable function by month 6. However, patient PROMIS data reflected persistent fatigue and low social participation, indicating the incomplete recovery of the quality of life beyond pain.

### 3.4. Case 4

An 85-year-old female with a history of aortic stenosis, HTN, DM, OA, and MM presented with worsening lower back pain after a fall. Imaging confirmed a new vertebral plana fracture at L3. An exam revealed tenderness at L3 and positive bilateral facet loading. The patient’s initial pain management included acetaminophen 500 mg twice daily as needed, acetaminophen/hydrocodone 10/325 mg three times daily as needed, and diagnostic medial branch injections at L2–3 and L3–4, which unfortunately provided minimal relief.

**Figure 8:**
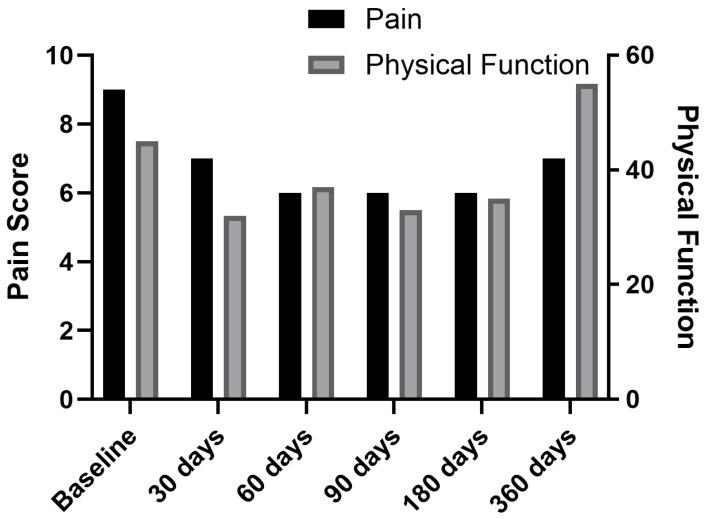
Change in pain scores and physical function scores over time for Case 4.

Two weeks later, the patient underwent fluoroscopy-guided PNS at L2 medial branch bilaterally. Pain decreased from 9/10 to 8/10 on day one. At 30 days, pain was 7/10, and by 60 days, it further reduced to 6/10. Leads were removed at 60 days, with reduced opioid use. Pain remained stable at 6/10 at 90 and 180 days but slightly increased to 7/10 at 360 days (Figure 3 and Figure 8).

This patient demonstrated moderate initial pain relief, which plateaued by 60 days and slightly increased by 12 months. Despite decreased opioid use, limited functional gains and minimal improvement in mood or fatigue were noted. These outcomes may reflect the progressive burden of multiple myeloma and systemic illness.

## 4. Discussion

In this case series, using PNS for managing vertebra plana fracture pain resulted in pain improvements and limited functional improvement as measured by PROMIS-29 scores (Table 2). Pain intensity and pain interference saw the largest improvements, with pain intensity dropping from 8.5 at baseline to 4.25 at one year and pain interference decreasing from 61.75 to 54.75 over the same period, exceeding the MCID of 2.0 points [21] listed in Table 3. These changes can be attributed to the direct effect of PNS in modulating nerve activity and interrupting pain signaling, which helps reduce the experience of pain and its interference with daily activities. Additionally, PNS’s possible opioid-sparing benefits may help avoid the negative side effects of pain medications, contributing to better overall function and less interference in patients’ lives.

Physical function based on PROMIS-29 scores improved slightly (38.5 at three months to 46.75 at one year). The early decrease could be attributed to the body adjusting to the treatment, postoperative recovery, or baseline physical limitations. Many patients may have had deconditioning or other comorbidities that initially limited their ability to regain function. However, as pain relief became sustained through PNS, patients experienced increased mobility and capacity to engage in activity, contributing to the gradual improvement in physical function. Pain reduction allows for a greater range of movement, less guarding, and a willingness to participate in rehabilitation efforts, which are crucial for improving long-term function. Domains which showed smaller changes included depression and anxiety, both of which are multifactorial, driven by long-standing psychological or social factors that PNS does not directly address. Additionally, chronic illness and comorbidities which are common in elderly patients with vertebral fractures may limit the extent of improvement in mood-related domains, even as physical symptoms improve.

VCF is complex as there are multiple sources of pain which exist: anteriorly due to vertebrogenic pain and posteriorly due to facetogenic pain [8,18]. Pain intensity is typically greatest in the initial weeks following a VCF, when patients experience decreased mobility. This increases the risk of deep vein thrombosis, constipation, and urinary tract infections [2,22,23]. Physical deconditioning can also develop, causing complications well after the fracture has resolved. For elderly patients with VCF who have failed conservative management with medications, physical therapy, and minimally invasive procedures such as medial branch or radiofrequency ablations, there is a demand for advanced interventions that bypass general anesthesia and provide quicker recovery, especially for those with heart conditions, due to the high risks of anesthesia-related complications [24]. While vertebral augmentation is an option, some patients may not be able to tolerate anesthesia. Hence, for patients with largely posterior VCF-related pain, medial branch PNS presents a promising therapeutic option by targeting pain transmission at the facet joints and surrounding musculature. Unlike radiofrequency ablation, which has been linked to multifidus atrophy [25], PNS may help preserve muscular integrity and prevent further spinal destabilization [26]. Maintaining paraspinal musculature is particularly beneficial for VCF patients, as muscle atrophy and instability can contribute to prolonged pain and disability. PNS cases are typically performed using only local anesthesia, reducing recovery time.

The mechanism of PNS works through the gate control theory and modulation of neurotransmitters, including increased GABA and glycine activity [27]. At the local level, increases in dopamine and serotonin levels are accompanied by decreased quantities of pro-inflammatory mediators. This is due to the frequent stimulation of A-delta and C fibers [27]. Evidence from rat models suggests that the change in the quantities of neurotransmitters can affect gene transcription and cause increased plasticity in NMDA receptors. These mechanisms give potential insight into why patients can experience lasting pain relief well after the completion of temporary treatment [28,29].

Recent studies have shown that PNS is effective for chronic low axial back pain that was otherwise refractory to radiofrequency ablation [29]. In another study, temporary PNS for chronic axial low back pain alleviated pain by over 30% at a 12-month follow-up [30]. Although these types of pain may have slightly different etiologies than VCF, the success of PNS in treating low back pain gives reason to explore PNS further in this space.

Short-term PNS treatment has been shown to provide pain relief for extended periods after the treatment ends. A retrospective study demonstrated that temporary PNS (for 60 days) was an effective treatment for various neuropathies, such as suprascapular neuropathy, chemotherapy-induced neuropathy, diabetic polyneuropathy, and radial neuropathy, with pain scores significantly reduced at 120 days post-treatment. Additionally, 50% of patients with diagnoses consistent with neuralgia or neuropathy experienced lasting relief after temporary PNS therapy [31]. It is important to note that while a 60-day temporary PNS was used in this case series, relief persisted even at the 12-month follow-up. While the continued benefit may be partly due to the lingering effects of PNS, it is also possible that the resolution of acute inflammation from the fracture played a role in the sustained pain relief.

Advancements in PNS systems have yielded options for brief and extended PNS trial periods. Some trials as short as 5–10 days have suggested that it is possible to achieve durable pain relief. In a study on orthopedic surgery, patients undergoing 14 days of PNS postoperatively experienced decreased pain scores and lower opioid requirements compared to those not receiving PNS [21]. Similar phenomena were observed in post-amputation patients receiving PNS treatment, as evidenced by less residual and phantom limb pain than in patients receiving only standard care [32,33,34].

Although rare, complications from PNS do exist. Risks include infection, bleeding, misplacement of electrodes, and refractory pain, with complications related to the hardware itself, such as lead migration being the most common. In a retrospective analysis of 89 patients, there were a total of five adverse events, with four of them being lead migration and one infection [31]. Well-designed randomized controlled trials are needed to further confirm the effectiveness of percutaneous PNS for various indications in pain management, including vertebral plana deformities.

This study also has several limitations. The small sample size limits generalizability and prevents robust statistical description. Furthermore, the absence of a control group hinders our ability to isolate the specific effects of PNS from natural recovery or placebo-related improvement. Patient heterogeneity—such as differences in age, comorbidities, fracture etiology (osteoporotic vs. pathologic), and baseline functional status—introduces potential confounding variables that may influence both treatment response and outcome trajectories.

Another key limitation is the reliance on subjective patient-reported outcome measures (PROMIS-29) without parallel objective assessments such as performance-based physical function tests or imaging-based evaluations. Although PROMIS-29 is validated and widely used in pain research, combining it with quantitative endpoints would enhance future evaluations.

Future research should focus on larger, multi-center randomized controlled trials with well-defined control arms, objective outcome measures, and stratification based on clinical subgroups to further elucidate the role and efficacy of PNS in this complex patient population.

## 5. Conclusions

This case series supports the use of PNS for patients with vertebral plana fractures who experience posterior VCF-related pain. PNS can significantly reduce pain and pain interference while improving physical function in patients with vertebra plana fractures. The placement of PNS devices was well-tolerated, with no procedural complications or adverse events reported. In each case, pain decreased by 33–75% on the pain scale, and all four patients reported a reduction in the use of narcotic pain medication. Based on this preliminary data, we propose large prospective studies to continue assessing PNS’s effectiveness in treating lower back pain associated with vertebral plana fractures.

## Figures and Tables

**Table 1:** Characteristics of patients who underwent temporary PNS implant for vertebra plana fracture **.

Patient	Age at PNS Procedure (y)	Sex	Level of Plana Compression	Pathologic vs. Osteoporotic Fracture	PNS Medial Nerve Target	Relevant Comorbidities	Percent Pain Relief
1	80	Female	L1	Osteoporotic	T12 bilateral	Coronary Artery Disease, Prior Cardiac Arrest	66%
2	71	Male	L4	Pathologic	L3 bilateral	Diabetes Mellitus, Rheumatoid Arthritis, Degenerative Disk Disease, Multiple Myeloma	75%
3	85	Female	L3	Osteoporotic	L2 bilateral	Coronary Artery Disease, Hypertension, DM, Osteoarthritis	60%
4	85	Female	L3	Pathologic	L2 bilateral	Hypertension, Diabetes Mellitus, Osteoarthritis, Multiple Myeloma	33%

L = lumbar vertebrae. ** = length of trial was 60 days.

**Table 2:** PROMIS-29 v2.0 mean (SD) of baseline and follow-up scores for four patients.

PROMIS-29 Domains	Baseline	30 Days	60 Days	3 Months	6 Months	1 Year
**Pain intensity (0–10)**	8.5 (0.6)	4.75 (1.7)	3.75 (1.7)	3.75 (1.7)	3.75 (1.7)	4.25 (1.9)
**Physical function**	50.5 (6.9)	44.75 (9.5)	40.25 (2.1)	38.5 (6.0)	40.25 (5.5)	46.75 (7.9)
**Anxiety**	71 (4.2)	71.5 (4.4)	69.75 (4.3)	69.5 (4.2)	70 (4.5)	69.25 (3.8)
**Depression**	69.25 (2.1)	65 (1.8)	64 (1.8)	69.25 (2.2)	65.5 (3.9)	68 (2.3)
**Fatigue**	68.5 (4.1)	70.25 (4.3)	67 (2.3)	67 (2.3)	65.25 (2.1)	65 (2.3)
**Ability to participate in social roles and activities**	50.75 (1.5)	48.25 (2.6)	48 (2.3)	49 (2.3)	48.75 (3.2)	48 (2.3)
**Pain interference**	61.75 (3.5)	56.25 (1.0)	55.5 (1.3)	55.5 (1.3)	54.75 (1.7)	54.75 (1.7)

**Table 3:** MCID analysis for PROMIS-29 v.20 mean domains for the four patients at 1 year.

PROMIS-29 Domains	Baseline Score	1-Year Score	Change Score	MCID	Meets MCID?
**Pain intensity (0–10)**	8.5	4.25	4.25	2.0	Yes
**Physical function**	50.5	46.75	3.75	3.0	Yes
**Anxiety**	71.0	69.25	1.75	3.0	No
**Depression**	69.25	68.0	1.25	3.0	No
**Fatigue**	68.5	65.0	3.5	3.0	Yes

## Data Availability

The original contributions presented in this study are included in the article. Further inquiries can be directed to the corresponding authors.

## Abbreviations

The following abbreviations are used in this manuscript:

CAD	Coronary artery disease
DM	Diabetes mellitus
HTN	Hypertension
MM	Multiple myeloma
MRI	Magnetic resonance imaging
OA	Osteoarthritis
PNS	Peripheral nerve stimulation
PROMIS	Patient-Reported Outcomes Measurement Information System
PRO	Patient-reported outcome
RFA	Radiofrequency ablation
VCF	Vertebral compression fracture
